# Genetics, genomics and breeding of fennel

**DOI:** 10.1186/s12870-025-06608-5

**Published:** 2025-05-07

**Authors:** Gabriele Magon, Fabio Palumbo, Gianni Barcaccia

**Affiliations:** Department of Agronomy, Food, Natural resources, Animals and Environment (DAFNAE), University of Padova, Campus of Agripolis, Viale Dell’Università 16, Legnaro, PD 35020 Italy

**Keywords:** Fennel, Biochemistry, Genetics resources, Molecular markers, Genomics, Breeding

## Abstract

Fennel (*Foeniculum vulgare* Mill. or *Anethum foeniculum*) stands out as a versatile herb whose cultivation spans across various regions worldwide, thanks to its adaptability to diverse climatic conditions. Its economic importance is mainly due to its numerous pharmaceutical properties and its widespread use in culinary applications. In this review, we first reviewed the chemical composition of this species, stressing the importance of two volatile compounds: t-anethole and estragole. The few cytological and genetic information available in the scientific literature were summarized. Regarding this latter aspect, we pointed out the almost complete absence of classical genetic studies, the lack of a chromosome-level reference genome, and the shortage of adequate transcriptomic studies. We also reviewed the main agronomic practices, with particular emphasis on breeding schemes aimed at the production of F1 hybrids and synthetic varieties. The few available studies on biotic and abiotic stresses were discussed too. Subsequently, we summarized the main studies on genetic diversity conducted in fennel and the available germplasm collections. Finally, we outlined an overview of the main in vitro regeneration techniques successfully applied in this species.

## Introduction

Fennel is an important crop native to the Mediterranean basin. It is specially adapted to the cultivation in well-drained, loamy soils, with a pH between 5.5 and 7.5, and it can tolerate a range of annual precipitations from 0.3 to 2.6 mm [31]. Due to its rusticity, fennel cultivation does not interest only its domestication center, but regards many other areas characterized by a subtropical and temperate climate, in which this species has been naturalized. Historically, fennel cultivation was accomplished for both food and medicinal purposes. The close bond between man and fennel dates back to the dawn of time. In early Sanskrit literature fennel was named as “*madhurika*”, yet 2.000 years BC. At the time of ancient Greece, it was considered a symbol of victory since, according to tradition, Marathon battle was fought in a fennel field. Indeed, the Greek name for this plant is “*μάραθο*” (màratho). Moreover, it was regarded by the Romans as a symbol of success, and its leaves were used to honor the winners in competitions. Mentioned as one of the nine plants in the tenth-century description of the occult'Nine Herbs Charm', fennel holds a significant place in ancient Anglo-Saxon tradition too. During the thirteenth century in England, fennel was esteemed as a royal spice and was presented to monarchs alongside fruits, bread, and delicacies such as pickled fish seasoned with fennel fruits, improperly called seeds [120].

Due to its versatility, fennel is employed worldwide in a very extensive range of culinary uses and cooking applications, involving soups, salads, spice, bakery, sausages, as well as non-alcoholic drinks and liqueurs. Nowadays, the major fennel producers are India, Pakistan, Italy, Russia, USA, Germany, and France [47]. India is the largest fennel producing country, with 90,000 hectares yielding 149,000 tons in 2017–2018. Over the past ten years, there has been an increase in the use of fennel and related spices in pharmacology and culinary. Due to factors like population growth, shifting dietary habits, and rising demand for value-added products like essential oil, fennel demand is expected to continue rising [94].

## Taxonomy

The taxonomic classification of fennel remains open. Originally, it was classified as *Foeniculum vulgare* Mill. [76], tribus Apieae, order Apiales, family Apiaceae. Fennel is also the only species of its genus. Two different subspecies are recognized: *F. vulgare* Mill. subsp. *piperitum* (bitter fennel, the wild form abundantly present in Mediterranean flora), and *F. vulgare* Mill. subsp. *vulgare*, which, in turn, includes var. *azoricum* (the Florence fennel, or “finocchio”, cultivated for its enlarged base), and var. *dulce* (grown prevalently for its fruits containing essential oil) [36, 93]. In a comprehensive phylogenetic analysis based on chloroplast DNA *rpl16* and *rpoc1* intron sequences and conducted on 147 species belonging to the Apiaceae family, fennel was classified as *Foeniculum vulgare* Mill. [32]. Two further studies, namely a phylogenetic analysis of 1,240 nuclear DNA ITS sequences performed on 959 species of the subfamily Apioideae [33] and a correlation study between the occurrence of expansion and contraction events of inverted repeat regions and the chloroplast-genome-based phylogeny of Apioideae [117], draw the same conclusions. Despite this, Frankiewicz et al. after a careful analysis of the factors influencing intrinsic and extrinsic diversification rate in the tribus Apieae, proposed to include the *Foeniculum*, *Schoenoselinum*, *Ridolfia* and *Pseudoridolfia* mono-species genera within the *Anethum* genus [41]. Fennel was therefore renamed *Anethum foeniculum*, as originally proposed by Linnaeus for its high content in anethole [105]. Fully aware of the ongoing taxonomic changes, to avoid confusion, we have nonetheless decided to adhere to the classical nomenclature, namely *Foeniculum vulgare*.

## Morphology and agronomic practices

Fennel is a perennial herb capable of growing up to 2 m. This species has finely divided, branching leaves and a smooth, polished stem that is light green, cylindrical, and upright. The finely divided, filiform (thread-like) segments of fennel leaves can reach a maximum length of 40 cm and a width of 0.5 mm. Inflorescences are represented by large and flat terminal umbels (5–15 cm broad) with 13–20 branches carrying 20–50 yellow golden hermaphrodite flowers on short pedicels. [47]. The fruit is an oblong achene, glabrous, with pronounced ribs and strongly aromatic. As a crop of mild growing conditions, fennel thrives in dry and cold weather, which increases fruit set. On the other hand, high temperatures cause early flowering and relatively low fruit output. The ideal temperature range for its growth is between 15 and 20 °C. Throughout the flowering period, the crop is vulnerable to frost conditions. Fennel can be cultivated as an intercrop or mixed crop because it is long lasting and owns a slow rate of growth at the beginning of the lifecycle. This feature, together with dormancy, was found to be responsible for a poor stability in production yields. To overcome this problem, 8–12 h of soaking are recommended to obtain a uniform germination [31]. Seeds can be placed directly in soil, or seedlings can be let germinate in a nursery and then transplanted. In this latter case, for one hectare, about 2.5 kg of seed are needed,On the contrary, 8–10 kg of seed are needed for direct sowing [74]. Since fennel seeds are tiny, line planting is mainly used. They should be sown no deeper than 1.5–2 cm, with a row spacing of 45–60 × 20–25 cm. Mid-September through the first week of October is the traditional planting season for fennel,for an early season crop, July is recommended for germination in nurseries, and August for transplanting. At 16–18 °C, seeds were observed to take 8–10 days to germinate [31]. The growing season is characterized by wide variations depending on the type of products and on fennel varieties. On a study conducted on fifty population of bitter fennel, three main groups were individuated based on their maturity habits: early, intermediate, and late maturing, requiring respectively 120, 175, and 230 days from seedling emergence to fruit harvest. Essential oil yield among the early-maturing fennels ranged from 2.5 to 104.6 L/ha/year (average 26.1 ± 0.1). The essential oil yield ranged from 10.1 to 152.2 L/ha/year (average 67.7 ± 1) in intermediate-maturing fennels and from 7.5 to 160.9 L/ha/year (average 46.4 ± 0.2) in late-maturing fennels [14]. Yield of fennel grown for lump purposes is reported to settle around 20 t/ha [27]. On the other hand, an average yield of 20–25 q/ha is described for fennel fruit production[31]. The recommended fertilization per hectare is 90 kg N, 40 kg P_2_O_5_ and 30 kg K_2_O to be applied as follow: at the time of sowing, 1/3 of the N and the entire dose of P_2_O_5_ and K_2_O; the remaining nitrogen should be supplied 30 and 60 days later. Three weeks after sowing, if the plant appears weak, an application of 1% urea could enhance fruit yield and essential oil content [11, 29, 31]. Biofertilization applications for improving the agronomic performances of this species were investigated too. Mishra et al. registered an increase in essential oil amount and fruit set thanks to the application of *Bacillus subtilis* strains PSB-1 and PBS-36, due to the natural phosphate solubilizing capacity specific to this bacterium [77]. Since these two inoculations were described to improve phosphate availability in semiarid and saline soil, this kind of application could be pivotal from a climate changes point of view, by boosting the effectiveness of nutrient utilization and decreasing the need for phosphate fertilizer application. Similar results were evidenced by the use of the arbuscular mycorrhiza *Glomus mosseae*, which was described to strongly improve quantitative and qualitative performances of fennel plants under drought stress conditions [122]. Fennel requires 200–350 mm of water either from natural rainfall or irrigation (0.6 IW/CPE), with the maximum grain yield observed at 10 days interval irrigation at a 100 plants/m^2^ planting density. Moreover, it is recommended to perform an organic matter amendment of 5 tons/ha if salt concentration of the irrigation water is higher than 1,000 ppm [27, 31].

## Chemical composition

*F. vulgare* is characterized by a complex mixture of bioactive compounds, including essential oils, phenolic compounds, and other secondary metabolites, which contribute to its various functional properties. The composition of fennel is influenced by multiple factors, such as genetic background, environmental conditions, and cultivation practices, leading to variations in its chemical profile.

### Minerals

The biochemical composition of fennel depends on cultivation conditions, soil composition and meteorological trend of the production season. In a study conducted on three different fennel cultivars spanning over two years, each of them with a double productive season (summer and autumn), pseudo bulb weight ranged from 199 to 383 g. Dry matter was described fluctuating from 61 to 76 g/kg, with a dietary fiber amount of 5.75, up to 7.59 g/kg. The mineral composition was observed varying in the following intervals: K from 4,241 to 5,851 mg/kg, Na from 77 to 512 mg/kg, Ca from 56 to 363 mg/kg and Mg from 82 to 389 mg/kg. Nitrates content ranged from a minimum of 650 to a maximum of 3,767 mg/kg. Finally, C vitamin content fluctuated from 87 to 347 mg/kg [65].

### Macronutrients

Regarding macronutrients, their content differs depending on the plant part considered. The highest moisture content can be detected in stems and leaves (76.36 and 77.46 g/100 g, respectively), while the lowest is found in the inflorescence (71.31 g/100 g). The most prevalent macronutrient in all the organs are carbohydrates. They exhibit an abundance of 18.44 to 22.82 g/100 g. The macronutrients that are less prevalent include proteins, reducing sugars, and fatty acids. The amount of proteins ranged from 1.08 g/100 g in stems to 1.37 g/100 g in inflorescences. Among the fennel tissues, the inflorescences and stems had the highest fat content (1.28 g/100 g) and, at the same time, the lowest amount of reducing sugars (1.49 g/100 g) [12]. More than twenty different fatty acids were individuated, with the unsaturated ones as the most abundant group showing a percentage varying from 66 to 80% of the total of fatty acids. Finally, the highest ω6/ω3 ratio was observed in inflorescences (2.20), the lowest in leaves (0.53) [16].

### Flavonoids and phenolic compounds

Flavonoid aglycones, flavonoid glycosides, and derivatives of hydroxyl cinnamic acid have been identified in fennel [90]. Total flavonoid content of hydroalcoholic extracts is about 12.3 ± 0.18 mg/g [37]. The fruit of this botanical specimen harbors a spectrum of compounds, including apigenin, quercetin, rosmarinic acid, cinnamic acid, hesperidin, 1,5 di-caffeoylquinic acid, ferulic acid, quercetin-7-*o*-glucoside, ferulic acid-7-*o*-glucoside, p-coumaric acid, caffeic acid, chlorogenic acid, gallic acid, and neochlorogenic acid [46]. Distinctly, chlorogenic acids, rosmarinic acid, 1,5-*O*-di-caffeoylquinic acid, 1,4-*O*-di-caffeoylquinic acid, 1,3-*O*-di-caffeoylquinic acid, 5-*O*-caffeoylquinic acid, 4-*O*-caffeoylquinic acid, and 3-*O*-caffeoylquinic acid, along with apigenin and quercetin, emerge as primary constituents of fennel fruits. Furthermore, it is noteworthy that the abundance of phenolic compounds surpasses that of flavonoids [37]. A quite recent study unveiled two novel compounds, specifically 3',8'-binaringenin and 3,4-dihydroxyphenethylalcohol-6-*O*-caffeoyl-*β*-*D*-glucopyranoside, isolated from wild fennel [42]. In addition, this plant is characterized by the prevalence of specific flavonoids, such as isorhamnetin-glucoside, kaempferol-3-arabinoside, kaempferol-3-glucuronide, quercetin-3-arabinoside, isoquercitin, and quercetin-3-glucuronide [70]. Moreover, aqueous extract of fennel highlighted presence of kaempferol-3-*O*-glucoside, kaempferol-3-*O*-rutinoside, and quercetin-3-*O*-galactoside [47].

### Volatile compounds

The essential oil derived from fennel has been documented to encompass at least 87 volatile compounds. The accumulation of the volatiles exhibits a degree of variability, manifesting ubiquitously across several anatomical components of the plant, including roots, stems, shoots, flowers, and fruits [12]. By comparing the accumulation profile of monoterpene hydrocarbons, oxygenated monoterpenes, and phenylpropanoids in immature, premature, mature, and fully mature fruits of Turkish sweet fennel (*Foeniculum vulgare* Mill. var. *dulce*), it was possible to conclude that the concentration of essential oil diminishes with the maturity. About the composition of fully mature fruits, the three main compounds observed are t*-*anethole (84.42–85.96%), estragole (4.22–5.16%) and limonene (2.96–3.86%) [111]. Interestingly, it has been demonstrated that the two most abundant compounds, namely t-anethole and estragole, derive from a common biosynthetic pathway ending with coumaryl acetate. This latter can be then converted into both t-anol and chavicol which, in turn, are respectively converted into t-anethole and estragole [43, 63].

Abdellaoui et al. [1] investigated the chemical composition of the essential oil extracted from the fruit of both wild and cultivated Moroccan fennel, suggesting that domestication processes did not enhance the essential oil yield [1]. As matter of fact, the greatest essential oil concentration (3.67%) was recorded in wild fennel, while the lowest (2.13%) was found in the cultivated one. The primary constituents of both types of fennel, based on chromatography measurements, were estragole (60.00% cultivated and 35.33% wild), t-anethole (22.15% cultivated and 52.27% wild), and fenchone (6.50% cultivated and 4.32% wild). The huge differences observed between and within wild and cultivated fennel in terms of volatile compounds content may be attributed to cultivation methods, phenological phase, distillation duration, genetic, regional, and environmental variables [14, 79, 81, 111]. For instance, in a study conducted on 12 different Iranian landraces of fennel (*F. vulgare* ssp. *vulgare*), the concentration of the single compounds in the total amount of essential oil varied tremendously. t-anethole varies from 1.24 to 88.45%, estragole from 0.22 to 59.1%, fenchone from 1.22 to 14.74% and limonene from 5.5 to 17.71% [14], outlining a situation in which seems very difficult to define a standardized normotype. The scientific literature on fennel is fraught with significant challenges, primarily due to the pervasive taxonomic confusion surrounding this species. The difficulty in accurately classifying the various subspecies and varieties has not only hindered botanical studies but also introduced substantial bias in research focusing on the accumulation of secondary metabolites, and thus essential oil. This confusion has often led to inconsistent or conflicting results in studies attempting to characterize the phytochemical profiles of the different subspecies, as shown earlier in this paragraph. Trying to make order in this controversial situation, it seems that *Foeniculum vulgare* ssp. *piperitum* consistently demonstrates low or negligible level of anethole, in favor of a higher concentration of estragole. This starks in contrast to *Foeniculum vulgare* ssp. *vulgare*, which is characterized by a high t-anethole and comparatively lower estragole levels. This differential pattern in volatiles compounds accumulation suggests a sort of metabolic trade-off which could be traceable to a common precursor shared between the two biosynthetic pathways [1, 3, 4, 53, 82, 85, 103]. By refining the systematic classification of fennel in this optic, it is clear that could be advantageous for the interpretation of the role of these secondary metabolites in terms of implications for adaptative differentiation between the subspecies, likely driven by ecological or selective pressure that prioritized the synthesis of specific volatiles compounds in response to environmental factors.

## Cytology and cytogenetics

Studies in fennel cytology and cytogenetics have contributed to understanding its genetic background and reproductive biology. These aspects are essential for its conservation, genetic improvement, and sustainable utilization in agriculture and medicine. The first approach regarded three different fennel varieties: *Foeniculum vulgare* var. UF32, *F. vulgare* var. UF31 and *F. vulgare* var. MSI, and it evidenced a good similarity rate in terms of chromosome number, size, and morphology among the three varieties. All of them highlighted a diploid somatic number of 2*n* = 22, with chromosome sizes spanning from 2.83 to 4.38 µm. The 4 C DNA amount in picograms detected was 18.93 ± 0.08, 18.44 ± 0.09, and 18.17 ± 0.11, respectively [28]. Contrasting results were found by Tomar et al. which reported a 2 C DNA content equal to 2.31 pg [113]. Similarly, in the study by Palumbo et al. the 2 C DNA content was estimated at around 2.64–2.86 pg [86].

Falistocco discovered some tetraploid fennels by analyzing individuals belonging to subsp. *piperitum* and subsp. *vulgare* (both vars. *azoricum* and *dulce*). From the chromosome counts, few seeds from the subsp. *piperitum* were found to be tetraploid (2*n* = 4*x* = 44) [36]. The number of tetraploids discovered in subsp. *vulgare* was even more in both var. *azoricum* and var. *dulce.* Also, the author identified pollen grains 1.25 times larger than normal ones. By analyzing the sporad constitution, it was found that these pollen grains were actually 2*n*, resulting from unreduced pollen maturation processes. All fennel plants producing larger pollen grains, in addition to regular tetrads, also showed dyads, which can lead to the formation of two 2*n* gametes. The production of 2*n* unreduced pollen grains was previously reported also by Sheidai et al. in a wild Iranian fennel population analysis [106]. The authors also suggested that, in addition to 2*n* pollen grains, unreduced 2*n* ovules could be produced in fennel and that polyploidy could represent a quite common occurrence rather than an occasional manifestation. The induction of polyploidy could represent a potential strategy for enhancing fennel agronomic performances by taking advantage of sexual polyploidization as useful tool for breeding initiatives.

## Genetic and omics resources in fennel

Even though fennel holds significance in global food culture due to its agronomic, nutritional, and pharmaceutical properties, researchers and breeders face the challenge of scarce biological and genomic data for this crop species. Indeed, in contrast to other crops from the *Apiaceae* family (e.g. carrot, celery, coriander), the investment in research worldwide appears notably inadequate. Consequently, the constrained progress in genetics and genomics research poses a barrier to advancing molecular assisted breeding programs and enhancing production on a global scale.

### Classical genetics studies

Classical genetics has been poorly investigated in fennel. Only 131 ‘gene’ sequences are available in GenBank, all derived from the assembly of the chloroplast genome (cpDNA, NC_029469.1, for further details, refer to the section on organellar genome sequencing). Additionally, the vast majority of the 242 ‘nucleotide’ sequences deposited in the same database, refer to sequences of mitochondrial, plastid (rbcL, matK, trnH-psbA), or nuclear origin (ITS1 and ITS2), produced exclusively for phylogenetic analyses within the Apiaceae family. Of particular interest was the identification of two key genes involved in the t-anethole biosynthetic pathway, namely phenylalanine ammonia-lyase (PAL, MK301446.1), and t-anol/isoeugenol synthase (IGS1, MK301447.1) [2].

Despite the importance of genetic linkage maps for investigating trait inheritance across the genome, fine mapping of quantitative trait loci (QTLs) or candidate genes, as of now, no genetic map based on molecular markers, isozymes or phenotypic traits has been produced in fennel.

The process of domesticating a species usually led to a significant reduction in genetic diversity and, consequently, breeders frequently turn to exotic germplasm to access desired genes. However, in the case of fennel, no study has ever been conducted on this matter. Despite the grey literature seems to confirm a potential infertility with the genus *Anethum*, experimental evidence is not currently available.

### Omics resources for fennel breeding

#### Nuclear genomics

A draft genome was released (GenBank, PHNY00000000) in 2018 [86]. Being the sequencing exclusively based on Illumina sequences (150 bp PE), it was possible to assemble approximately 75% of the estimated fennel haploid genome (1.01 out of 1.37 Gb). Consistently, BUSCO analysis revealed a completeness of 78.2%, with 72% of the identified genes present in single copy. The draft genome has been recently used to study its MiRnome and to assess its impact on the transcriptomes of *Arabidopsis thaliana* and *Homo sapiens* [115]. One hundred fennel miRNAs and their target genes, encompassing 2,536 genes in *Homo sapiens* and 1,314 genes in *Arabidopsis thaliana* were identified.

The bioinformatic analysis of the repetitive regions of the draft genome allowed also the identification of > 103,300 SSR elements. A subset of 100 microsatellites with dinucleotide and trinucleotide repeat motifs and a motif length of ≥ 25 repeats was randomly selected for preliminary testing. Out of these, 27 SSR markers were efficiently arranged into five PCR multiplex assays and validated using a core collection of 100 fennel individuals potentially beneficial for the cultivation of inbred lines and the development of F1 hybrids. The same markers were subsequently tested on a set of 20 fennel germplasm lines from India demonstrating an intraspecific transferability of around 50% [67].

Although the lack of a physical map makes comparative genomic approaches impossible, an alternative system for the development of molecular markers is the use of heterologous primers. Before the release of the fennel draft genome, carrot was the sole apiaceous plant with an available genome assembly [54] and a robust set of SSRs [23]. Typically, SSR primers transferability from carrot to other species within the same family was the prevailing approach. In this context, Aiello et al. [5] assessed the transferability of SSR markers from carrot to fennel, but out of 39 SSR markers, only 23% of them were found to be suitable for fennel. Similarly, Kumar et al. [69] investigated the transferability of 12 SSR primer pairs originally developed in *Anethum sowa* (dill seed) to fennel, with significantly more encouraging results: the frequency of clear and strong bands resulted in 83.3%.

#### Organellar genomics

**Fig. 1:**
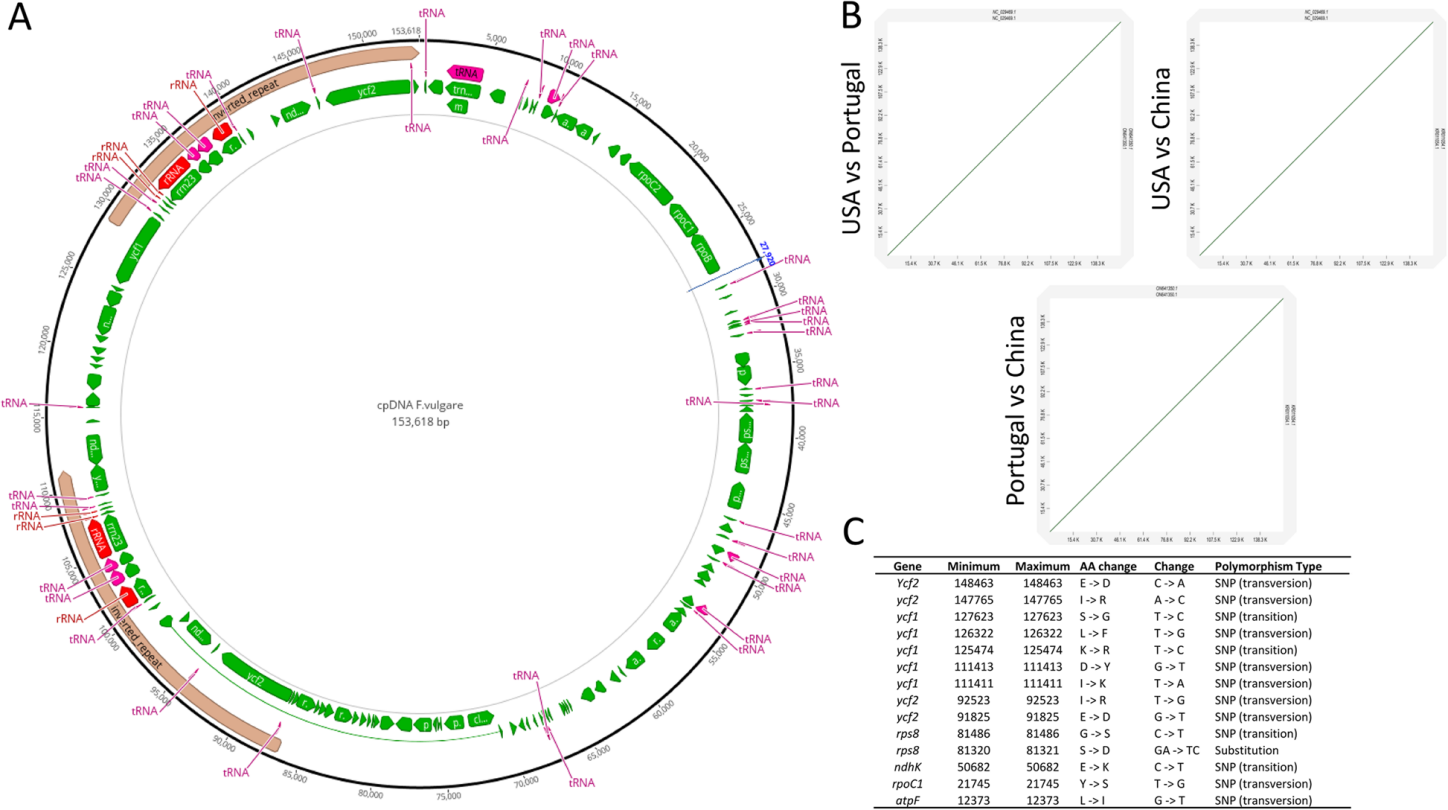
**A** *Foeniculum vulgare* plastidial genome representation (GenBank accession ON641350.1); this panel has been drawn by uploading in Geneious 11.1.4 software (http://www.geneious.com) the cpDNA fasta sequence and its corresponding GFF3 file. **B** Dot plot analysis among three cpDNA fennel accessions (i.e., NC_029469.1 for USA, KR011054.1 for China, and ON641350.1 for Portugal); the collinearity analysis was performed through the online tool D-genies [22] and the Minimap2 v2.26 aligner. **C** List of 14 non-synonymous SNVs (Single Nucleotide Variants) found aligning the five-cpDNA sequences available in GenBank for fennel (i.e. NC_029469.1, OM307067.1, ON641373.1, ON641350.1, ON641289.1). The alignment was performed using LASTZ at default parameters whereas SNV calling (minimum variant frequency = 0.2) was achieved in Geneious 11.1.4 software

Plastids and plastid DNA (cpDNA) stand out as one of the primaries defining features of plant cells. While their central role involves facilitating photosynthesis, plastids also host crucial cellular processes such as starch, fatty acid, pigment, and amino acid synthesis [118]. cpDNA also represents a valuable tool for elucidating systematic relationships within genera and species. To achieve this goal, it was suggested employing *rbcL* and *matK*, along with additional informative loci such as the intergenic spacer *trnH-psbA* and the *trnL* intron [87]. Fennel is among the 366 species belonging to the Apiaceae family for which cpDNA has been sequenced, assembled, annotated and deposited in GenBank. In reality, five different plastidial genomes are available in GenBank for this species (NC_029469.1, OM307067.1, ON641373.1, ON641350.1, ON641289.1) ranging from 153,618 bp to 153,665 bp. The complete cpDNA of fennel follows the typical quadripartite structure, comprising a large single-copy (LSC) region of around 86,650 bp, a small single-copy (SSC) region of around 17,470 bp, and a pair of inverted repeats (IR) regions of around 24,750 bp each. Within the chloroplast genome sequence, there are 130 genes, including 85 encoding genes, 37 transfer RNA genes, and 8 ribosomal RNA genes [119]. In Fig. 1A the structure of one of the cpDNAs available in GenBank (i.e. ON641350.1) is depicted.

The Figure has been drawn by uploading in Geneious 11.1.4 software (http://www.geneious.com) the cpDNA fast sequence and its corresponding GFF3 file (with all the annotations), both retrieved from GenBank. Despite submitted by three different research groups from as many different geographical areas, namely University of Illinois (USA, NC_029469.1), the Third Affiliated Hospital of Nanchang University (China, OM307067.1) and Instituto de Biologia Experimental e Tecnologica (Portugal, ON641373.1, ON641350.1, ON641289.1) the five cpDNAs resulted perfectly collinear and free from any rearrangement event. Indeed, using the online tool D-genies [22] and the Minimap2 v2.26 aligner, we conducted basic analyses of collinearity among three representative cpDNA genomes of as many geographical origins (i.e., NC_029469.1 for USA, KR011054.1 for China, and ON641350.1 for Portugal Fig. 1B). Even from the analysis of SNVs (Single Nucleotide Variants), the five-cpDNA sequences were found to be extremely conserved. The fasta sequences of the five cpDNA were first aligned using LASTZ at default parameters and then used for SNV calling (minimum variant frequency = 0.2, in Geneious 11.1.4 software). One-hundred and thirty intraspecific SNVs were identified (0,08% of polymorphic positions) of which only 25 falling in coding regions, and 14 produced amino acid changes (Fig. 1C).

Mitochondria and mtDNA play a crucial role in in the metabolism of plant cells, providing energy and metabolic intermediates. Along with *Daucus carota*, *Dystaenia ibukiensis*, *Dystaenia takesimana*, *Oenanthe javanica*, *Cuminum cyminum*, *Apium gravelones*, *Apium leptophyllum*, *Bupleurum falcatum*, *Bupleurum chinense*, *Ferula sinkiangensis*, *Coriandrum sativum*, *Saposhnikovia divaricate*, fennel is one of the 13 species of the Apiaceae family (≈3820 species) for which a mitochondrial genome is available [89]. According to the authors, the mtDNA molecule resulted of 296,483 bp and contained 24 mtDNA genes, 26 ORFs, 25 tRNAs, and 9 rRNAs. Specifically, the mitochondrial DNA (mtDNA) of a cytoplasmic male sterile (CMS) individual was assembled, and through comparison with its male fertile counterpart, a putative candidate gene potentially involved in the lack of pollen production was identified. The CMS and MF differed in the *ATP6* sequence, a key component of the transmembrane F_o_ portion of the ATP synthase which, in other species (including carrot [110]) has been already associated with CMS phenomena. In particular, the CMS mtDNA exhibited a 300 bp truncation at the 5’-end. Based on these observations, the energy deficiency model could elucidate the CMS behavior. As per this theory, the partial or complete dysfunction of one or more subunits within the mitochondrial electron transport chain (complexes I-IV) or ATP synthase (complex V) can lead to mitochondrial deficiencies [24].

#### Transcriptomics

The first available fennel transcriptomic resource is a leaf transcriptome produced in 2018 and constituted by 79,263 transcripts [88]. Through orthology analysis, it was possible to identify eleven putative enzyme sequences involved in the biosynthesis of t-anethole. Moreover, 6,411 Expressed Sequence Tag-SSRs (EST-SSR), predominantly falling into the di- and tri-nucleotide categories, were also identified, but not validated. Despite being generally less polymorphic than extra genic SSRs, EST-SSRs exhibit enhanced amplification success in related species, proving valuable for evaluating functional diversity and facilitating marker-assisted selection. Additionally, they serve as anchor markers for evolutionary and comparative mapping studies, given their greater transferability among related species [45]. Finally, a catalog of 43,237 SNPs and 3,955 indels variants discriminating four inbred lines was produced in the frame of the study. Except for this study, the identification of SNV through NGS platforms in fennel has never been taken into consideration.

More recently, the anthocyanin coloration and aroma formation in purple and green fennel were systematically studied by integrating transcriptomics and metabolomics approaches. The critical genes associated with the biosynthesis and regulation of anthocyanins and volatile phenylpropanoids were isolated and studied carefully in transiently transfected tobacco cells and transgenic tomato plants [123].

Finally, one last RNA-seq analysis was performed on fennel leaf tissues to identify differentially expressed genes responsive to elevated concentration of CO_2_ (eCO_2_). This peculiar study demonstrated that the growth and antioxidant activity of fennel seem to benefit from eCO_2_ [56]

## Conventional and marker assisted breeding in fennel

### Development of F1 hybrid varieties

Fennel exhibits hermaphrodite flowers with a tendency towards protandry (i.e. male reproductive organs mature before the female ones), leading to a predominantly outcrossing behavior [66]. Leveraging heterosis in various outcrossing crops has enabled breeding to achieve significant production enhancements. Indeed, heterosis or hybrid vigor is defined as an increase in the agronomic value of F1 hybrids when compared to the average value of both parents. Moreover, hybrids are particularly appreciated for the uniformity of the crop and less variation in maturity. As of today, the majority of fennel varieties are F1 hybrids, as evidenced by the flagship products of leading seed companies in the global market (e.g. [17, 19, 35]). Nonetheless, effective pollination control mechanisms, particularly male sterility, are necessary for hybrid breeding. Because of the economic importance associated with pollination control mechanisms, breeding companies often keep most of the information confidential, resulting in limited information on sources of male sterility. According to the few information found in the literature, cytoplasmic male sterility (CMS) is available and used for the production of F1 hybrids in fennel and few other Apiaceae species namely *Daucus carota*, *Apium graveolens*, and *Pastinaca sativa* [57, 72, 73, 89]. Given the absence of any self-incompatibility mechanism [107] and considering the complex morphology and size of the floral umbel (which makes manual emasculation unfeasible on a large scale), the use of CMS seems the only viable option for F1 hybrid production in fennel.

**Fig. 2:**
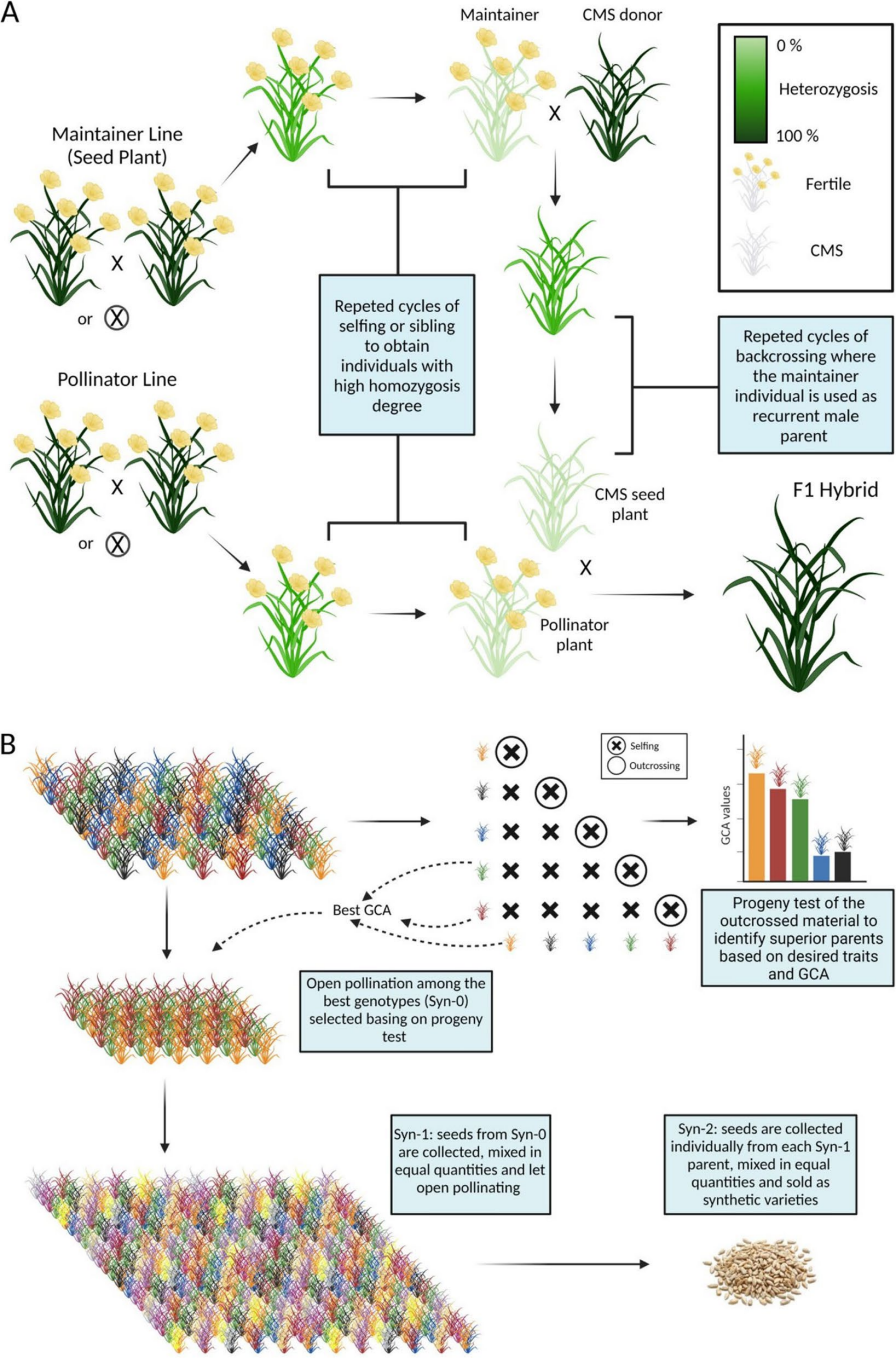
Simplified breeding schemes describing the development of F1 hybrids through the exploitation of a cytoplasmic male sterility (CMS, **A**) and the constitution of synthetic varieties (**B**) in fennel

Overall F1 hybrids development through the use of male-sterility is laborious, time-consuming and requires at least five main steps (Fig. 2A) [87]:

- introgressing the CMS trait into the future seed parent lines (or female lines);
- maintaining the CMS lines over generations using nearly isogenic male-fertile maintainers;
- producing (preferentially through cycles of selfing or sibling) highly homozygous inbred lines, to be used for the production of F1 hybrids;
- planning a number of diallel or half-diallel crosses among superior inbred lines;
- evaluating the F1 hybrid offspring resulting from each diallel or half-diallel cross to assess the general combining ability (GCA) and specific combining ability (SCA) of the parents. Parents exhibiting high average combining ability across crosses are regarded as possessing good GCA, while those whose ability to combine effectively is limited to specific crosses are considered to have good SCA [84].

Molecular tools can significantly accelerate and enhance the entire process. Single nucleotide polymorphisms (SNPs) and simple sequence repeats (SSRs) are particularly advantageous due to their reproducibility, codominant nature, locus specificity, and random distribution across the genome. In this context, molecular markers can be of great help in verifying the isogenicity between CMS and related maintainer line, predicting the homozygosity/uniformity of the inbred parent lines and the heterozygosity of the resulting offspring, and estimating the genetic distance among parental inbred lines in order to plan targeted crosses (avoiding expensive full diallel crosses) [48].

Despite this, studies focused on marker-assisted breeding (MAB) programs aimed at F1 hybrid production in fennel remain very limited. Palumbo et al. used SSR data to determine i) the level of homozygosity within each individual inbred line, guiding potential additional selfing or sibling cycles, and ii) the degree of genetic similarity across all pairwise comparisons between parental inbred lines, aiding in the identification of the most divergent combinations and the formation of experimental F1 hybrids [86]. In a second study, Scariolo et al. genotyped 8 breeding populations, each constituted by CMS lines (seed plants), their maintainers, pollinators (pollen donor) and the resulting F1 hybrid progenies to assess the uniformity of each line and to identify any correlation between the stability of the hybrids and the genomic background of their parents [99].

One critical aspect in F1 hybrid constitution is the production of inbred lines. Traditionally, this is achieved through selfing, which requires multiple generation cycles and the need for plant isolation. In this context, phenomena of inbreeding depression can be observed [62]. This latter aspect has been recently deepened in fennel by Shojaiefar et al. [107]. Fennel progenies resulting from selfing exhibited a fruit yield that was 20.86% lower than that resulting from bee pollination in the first year, probably due to a reduction in the number of effective umbels per plant. Saeidnia et al. also reported a decrease in fruit productivity, essential oil yield, β-myrcene, and limonene in selfed plants when compared to open pollinated (OP) populations [96]. Similarly, Hosseini et al. reported different levels of inbreeding depression in a comparison between OP and selfed fennel plants [50]. To overcome the challenges associated with the production of inbred lines (primarily, lengthy timelines and inbreeding depression), homozygous plants can be obtained through haploid induction. Haploid plants possess a gametic chromosome number in their sporophytes, and when their genome is duplicated, doubled haploids (DH) are produced. DHs have three significant advantages for plant breeding: (i the production of true homozygous plants (100%; (ii the time required to obtain homozygous plants (typically, one generation is significantly shorter compared to inbreeding (which may take several generations; and (iii the possibility to reveal and fix recessive traits that would otherwise remain hidden in heterozygous individuals [62]. Protocols based on anther or microspore culture for haploids and doubled haploids production are available in the scientific literature for fennel [101]. Evidences of doubled haploid production and field testing can be found in two studies by Ferrie et al. [38, 39].

### Development of synthetic varieties

In the absence of male sterility systems, the development of synthetic cultivars can serve as a viable alternative for harnessing heterosis. Synthetic cultivars are typically created through the random mating of a limited number of components (typically, three to five) selected based on their combining ability and yield performance [7]. While synthetic cultivars may not harness heterosis to the same extent as F1 hybrid cultivars, they offer numerous other advantages. For instance, they do not require meticulous pollination control, are often more cost-effective and easier to develop. Furthermore, synthetic cultivars boast higher genetic diversity, resulting in a more consistent performance under variable growing conditions and serving as a valuable gene pool for breeding endeavors [18]. Key stages in the development of synthetic cultivars include (Fig. 2B) [13, 116]:

- breeding material (e.g. inbred lines) is placed in a testcross (polycross or topcross) to produce seeds by random pollination;
- yield test of the outcrossed material to identify superior parents (Syn-0) based on desired traits and GCA;
- open pollination among the Syn-0 parents
- seeds (Syn-1) are collected individually from each (Syn-0) parent and are combined in equal quantities
- open pollination among the syn1 plants
- seeds (Syn-2) are collected individually from each Syn-1 parent, are mixed in equal quantities and placed on the market.

Similar to hybrids, the development process of synthetic varieties can also be streamlined with the use of molecular approaches. Bahmani et al. selected and allowed naturally cross-pollinate elite parents (syn-0) exhibiting superior GCA for either essential oil content or fruit yield in drought stress conditions. Following the development of the initial generation of synthetic cultivars (Syn-1), their performance was assessed in comparison to their related elite parents under both well-irrigated and drought conditions. Overall, five synthetic cultivars demonstrated higher essential oil and fruit yields compared to their parental lines under drought stress [13]. The same group also investigated the antioxidant activity, total phenol content, and phenolic compounds available in three synthetic cultivars and seven parental populations of fennel to demonstrate the potential of the former over the latter [7]. Despite this, there is no study focused on DNA marker-assisted breeding (MAB) programs aimed at synthetic lines constitution in fennel.

### Studies on abiotic and biotic stress

Drought stress is a significant abiotic factor that diminishes plant productivity, leading to a decline in photosynthetic activity, heightened oxidative stress, changes in cell wall flexibility, accumulation of abscisic acid and the production of toxic metabolites [61]. The drought stress seems to have a particularly impactful effect on fennel. Exposure to drought resulted in decreased plant height, branch number, and fruit and oil yield [78]. It was also found that drought stress during seed development resulted in a reduction of both the fruit yield and quality of fennel fruits in terms of aromatic oil content [61, 91]. Furthermore, Seidnia et al. demonstrated that water deficit greatly decreased grain yield and α-pinene, t-anethole, and anisaldehyde content [96]. Drought and salt stress are tightly interconnected: irregular or reduced irrigation can also cause salinity stress with reduced yield and product quality. For example, physiological measurements from Attia et al. suggest that salt-treated seedlings experience limitations in germination, growth, mineral uptake, and membrane damage [10].

To enhance both the quality and quantity of fennel yields, it is imperative to develop drought-and/or salt-tolerant genotypes. Few attempts in this direction were made in the last few years. Hosseini et al. found that some physiological parameters such us plant height, number of umbels per plant, seed width, seed length and thousand seed weight are closely linked to drought tolerance, making them valuable candidates for inclusion in a selection index aimed at improving fruit yield and identifying drought tolerant genotypes. Based on this correlation they were able to identify superior genotypes within synthetic and OP populations exhibiting higher seed production, yield stability, and drought tolerance, making them suitable candidates for future breeding programs [50]. The same authors analyzed the variability, stability, persistence, and recovery post-drought of numerous fennel genotypes sourced globally, identifying and selecting superior genotypes for potential future investigations [51]. Moreover, Shafeiee and Ehsanzadeh studied the physiological and phenotypical responses of six Iranian *F. vulgare* genotypes subjected to five water solution with increasing amount of NaCl (i.e. 0, 30, 60, 90, and 120 mM). Results revealed a modest salinity tolerance for three genotypes [102].

As regards biotic stresses, in 2020 Aiello et al. comprehensively reviewed the main fungal-origin pathologies affecting fennel, especially downy mildew, powdery mildew, and collar rot [6]. Instead, little is known regarding bacteria-origin diseases affecting fennel and *Erwinia caratovora* and *Psuedomonas syringae* pv *apii* represent the two only species mentioned in the scientific literature [60]. Tolerant genotypes and genetic determinants responsible for resistances are never mentioned in the literature.

## Germplasm and genetic diversity studies

Analyzing the genetic variation in germplasm collections is crucial not only for safeguarding local varieties but also for accurately classifying genetic material and pinpointing core accession subsets that could be valuable in plant improvement. This process also aids in comprehending the inheritance patterns of various traits, providing valuable insights for breeding programs [51]. Publicly available collections for fennel are extremely limited. Overall, most of the studies assessing the genetic diversity of fennel germplasm, were based on private, local and, often, inaccessible collections (Table 1). The main collaborative program facilitating the long-term conservation and the utilization of plant genetic resources (including fennel) is represented by the ECPGR (European Cooperative Programme for Plant Genetic Resources). Within the ECPGR, the European Umbellifer database (EUDB) was established in 1997 and underwent revisions by the Umbellifer Working Group in 2013. Its main role is to coordinate activities among the different member states interested in safeguarding the genetic resources of 9 *Apiaceae* genera, notably *Anethum* L. (dill), *Apium* L. (celery), *Carum* L. (caraway), *Chaerophyllum* L. (chervil), *Coriandrum* L. (coriander), *Daucus* L. (carrot), *Foeniculum* Mill. (fennel), *Pastinaca* L. (parsnip), and *Petroselinum* Hoffm. (parsley) (The European Cooperative Programme for Plant Genetic Resources [112]. According to the EUDB, recently updated integrating data deriving from EURISCO (European Search Catalogue for Plant Genetic Resources) and national programs, there are 740 accessions of fennel, many of which were originally collected in Portugal (126), Israel (75) and Italy (53) [64]. On the other hand, The Leibniz Institute of Plant Genetics and Crop Plant Research (Gatersleben, Germany), holds the biggest collection of fennel accessions (148 from 32 countries).

**Table 1:** Genetic diversity studies carried out in fennel germplasms. For each study the geographical origin, the number of samples tested, the type of morphological measurements, biochemical analyses and molecular markers are indicated

Author (year)	Geographical origin (N)	Morphological measurements	Biochemical analyses	Molecular markers
(Bernáth et al. 1996 [21])	Italy (4), France (1), Hungary (1); Korea (1), Belgium (1), unknown (5)	P H, leaf mass, F size, thousand F mass	t-anethole, fenchone, limonene, methyl chavicol, α-pinene, β-pinene	
(Piccaglia and Marotti, 2001 [92])	Italy (13)	P H, stems pp, Us pp, P W, P W of stems, leaves, and Us	Fruit oil content, α-pinene, camphene, sabinene, β-pinene, myrcene, α-phellandrene, hexyl acetate, δ−3-carene, p-cymene, limonene, c-ocimene, t-ocimene, γ-terpinene, fenchone, camphor, terpinen-4-ol, methyl chavicol, t-carveol, carvone, anisaldehyde, t-anethole	
(Singh et al. 2003 [108])	India (34)	F yield pp, Fs per U, U diameter, N. Us pp, N. primary, branches pp, N. secondary branches pp, P H, 100-S W		
(Lal et al. 2006 [71])	India (37)	DT flowering, P H, Us pp, diameter of main stem, Us on main stalk, F yield/plot,	Fruit oil content, t-anethole	
(Zahid et al. 2009 [121])	Pakistan (50)	Germination percentage, DT flowering, p H, stem girth, nodal distance, U diameter, DT 50% maturity, DT harvesting, F yield per row, 100-F W and harvest index		RAPD (24 primers)
(Meena et al. 2010 [75])	India (13)	P H, N. of primary branches, angle of primary branches, L of lower node of stem from ground surface, L of upper node of stem from ground surface, L of middle node of stem from ground surface, diameter of U, U pp, umbellate/U, F/U, P W, yield/plot		
(Napoli et al. 2010 [83])	Italy (56)		78 compounds identified	
(Grover et al. 2011 [44])	India (7)			RAPD (7 primers)
(Bahmani et al. 2013 [15])	Iran (25)			RAPD (10 primers)
(Kelardashti Maghsoudi et al. 2015 [58])	Iran (55)	P H, flowering date, flower diameter, DT 100% flowering, N. lateral shoots, dry W pp, F yield	Leaf oil content	SRAP (12 primer combinations)
(Saxena et al. 2016 [97])	India (91)		α-pinene, camphene, β-pinene, myrcene, cymene, γ-terpinene, 4-allyl anisole/methyl chavicol or estragol, anethole, geranyl acetate and p-anisaldehyde	
(Choudhary et al. 2018 [26])	India (17)			RAPD (16 primers), ISSR (10 primers)
(Hosseini et al. 2021 [49])	Iran (19), Belgium (2), Cyprus (1), Germany (2), Cuba (2), Portugal (1), Ethiopia (1), Pakistan (1), USA (1); Hungary (2), Egypt (1)	DT 50% flowering, DT 90% maturity, P H at 50% maturity, P fresh W, P dry W, N. Us pp, N. umbellets per U, N. Fs per umbellet, F yield, harvest index, 1000-S W	Fruit oil content, fenchone, limonene, estragole, t-anethole	
(Križman and Jakše, 2022 [68])	Slovenia and Croazia (155)		Estragole, t-anethole, fenchone	RFLP (on ITS region)
(Khalil et al. 2023 [59])	Pakistan (30)	P H, N. Us pp, U diameter, rays produced/U, fruits produced/umble, fruit color, fruit shape		
(Deshwal et al. 2023 [30])	India (216)	DT 50% flowering, DT maturity, P H, branches pp, Us pp, umbellets per U, Fs per U, P W, F yield pp		

*pp* per plant, N Number of, *P* Plant, *F* Fruit, *U* Umbel, *DT* Days to, *W* Weight, *L* Length, *H* Height

Despite its potential, there is no comprehensive study assessing the genetic diversity of this germplasm. Overall, apart from a couple of studies [21, 49], the few studies available in the literature often refer to a limited number of local varieties (often landraces) all collected from the same country. Most of the studies were carried out in India, Pakistan, Italy, and Iran, world leaders in fennel production (Table 1). Additionally, it is worth noting the complete lack of standard varieties, which could improve comparability across different studies.

Also, a significant portion of the available studies is based on morphological markers, sometimes combined with biochemical markers (i.e., chemical composition of the oils). Both type of markers are susceptible to phenotypic plasticity (being highly influenced by the environment) and can vary significantly depending on the tissue and stage of development [25, 80]. On the contrary, molecular markers present various benefits compared to traditional, phenotype-based methods, being consistent and detectable across all tissues irrespective of cellular growth, differentiation, development, or defense status. Moreover, they are not influenced by environmental factors [80]. Despite the availability of codominant SSR, EST-SSR, and SNP tools [5, 69, 86, 88], the molecular markers used in the reviewed studies were always dominant, mostly RAPD, and to a much lesser extent, SRAP, RFLP and ISSR markers [15, 44, 49, 58, 68, 121]. However, the reproducibility of RAPD e SRAP profiles is very problematic [8] and the use of co-dominant markers (e.g., SNPs or SSR) in future studies is recommended **(**Table 1**)**.

## In vitro techniques for plant regeneration

Advancements in functional genomics research in *F. vulgare* are closely linked to the development of reliable and efficient regeneration methods under in vitro conditions. Over the years, a wide array of tissue culture approaches has been optimized for this species, supporting both organogenic and somatic embryogenic pathways. Initial efforts focused on the induction of somatic embryos from diverse explant types—such as shoot apices, immature floral structures, cotyledons, leaves, and hypocotyls—using specific hormonal regimes involving auxins (e.g., 2,4-D, NAA) in combination with cytokinins (BAP or kinetin). Although early observations pointed mainly to limited bud proliferation through ramification, subsequent studies revealed the potential for direct shoot regeneration (caulogenesis) and somatic embryogenesis from callus, particularly in selected genotypes like Francia Pernod and certain Turkish accessions when exposed to optimal hormonal balances [9, 95, 109, 114]. A significant milestone was reached with the establishment of a highly responsive embryogenic system based on immature flower explants [40]. Comparative analyses between plants regenerated via organogenesis and those derived from somatic embryos further supported the genetic stability and uniformity of regenerated lines [20]. Shoot induction was also successfully accomplished from embryonic and shoot tip explants, especially when cultured on media enriched with BAP and IBA [55, 98, 104]. Interestingly, the inclusion of specific micronutrients, particularly copper and zinc, has been shown to enhance morphogenetic efficiency in fennel [34]. In parallel, liquid cultures derived from calli of bitter fennel have been employed to establish suspension cultures capable of both regenerating plantlets and producing valuable secondary metabolites such as trans-anethole [52]. Furthermore, protocols enabling the generation of haploid and doubled haploid plants through anther and microspore culture have been reported [39, 100, 101], offering crucial biotechnological tools for future applications in genetic transformation and functional gene analysis in this crop.

## Future perspectives

In this review, we highlighted several knowledge gaps in fennel that—we are confident—will inspire future research endeavours. Despite its economic and cultural significance, a chromosome-level genome assembly is lacking and comprehensive transcriptomic studies focusing on fennel developmental processes are scarce. The lack of such investigations limits our understanding of gene expression dynamics throughout its growth stages and hinders the identification of key regulatory mechanisms. Also, the genetic basis underlying fennel resistance to biotic and abiotic stresses remains largely unexplored. Studies deciphering the genes involved in stress responses are crucial for developing resilient cultivars capable of withstanding environmental challenges. To the best of our knowledge, genetic engineering and genome editing techniques have never been employed in fennel. The absence of research in this area represents a significant gap in harnessing genetic manipulation for crop improvement, limiting opportunities for targeted trait modification. From a productive point of view, agronomic improvements of fennel predominantly rely on conventional and suboptimal breeding programs, but there is potential for progress through innovative molecular approaches. In this context, molecular tools hold immense potential for fennel breeding although their utilization remains underexplored. Finally, yet importantly, a comprehensive assessment of genetic diversity within worldwide fennel germplasm is needed. Understanding the genetic variability of a species is pivotal for broadening the genetic base of breeding programs and facilitating the development of superior cultivars with enhanced adaptability and productivity. In summary, addressing these research gaps through concerted efforts in genomics, transcriptomics, functional genomics, and breeding methodologies is imperative for unlocking the full potential of fennel and ensuring its sustainable cultivation in the face of evolving agricultural challenges.

## Data Availability

No datasets were generated or analysed during the current study.
